# Pituitary metastasis as an endocrine–neuro-ophthalmologic emergency: clinical red flags and outcomes from a contemporary tertiary-center series

**DOI:** 10.1007/s11102-026-01715-4

**Published:** 2026-07-01

**Authors:** José Calixto Llumiquinga Marcayata, Jacqueline Miyuki Viel, Matheo Augusto Morandi Stumpf, Bibiana de Souza Boger, Matheus Moreli Porceban, Mario Padula, Ericka Barbosa Trarbach, Marilena Nakaguma, Malebranche Berardo Carneiro Cunha-Neto, Rafael Loch Batista

**Affiliations:** 1 Unidade de Neuroendocrinologia, Disciplina de Endocrinologia e Metabologia, Hospital das Clínicas, Faculdade de Medicina da Universidade de São Paulo, São Paulo, Brasil; 2 Divisão de Neurocirurgia, Hospital das Clínicas, Faculdade de Medicina da Universidade de São Paulo, São Paulo, Brasil; 3 Departamento de Radiologia, Hospital das Clínicas, Faculdade de Medicina da Universidade de São Paulo, São Paulo, Brasil; 4 Divisão de Oncologia Endócrina, Instituto do Câncer do Estado de São Paulo (ICESP), Faculdade de Medicina da Universidade de São Paulo, R. Dr. Ovídio Pires de Campos, 225 - Cerqueira César, São Paulo, Brasil; 5 Developmental Endocrinology Unit, Division of Endocrinology and Metabolism, LIM/42, Universidade de Sao Paulo, Sao Paulo, 05403-900 Brazil

**Keywords:** Pituitary metastasis, Sellar lesion, Neuro-oncology, Arginine vasopressin deficiency, Pituitary adenoma, Pituitary carcinoma, Pituitary neuroendocrine tumors

## Abstract

**Purpose:**

To characterize the clinical presentation, endocrine phenotype, and outcomes of pituitary metastasis in a contemporary tertiary-center cohort, with emphasis on clinically actionable diagnostic red flags.

**Methods:**

We conducted a retrospective single-center case series of 14 consecutive patients diagnosed with pituitary metastasis between 2010 and 2025. Presenting manifestations, endocrine features, radiologic findings, primary tumor origin, treatment patterns, and overall survival were assessed. In patients without histopathologic confirmation, diagnosis was established using the validated clinicoradiologic model proposed by Yuzkan et al.

**Results:**

Median age was 52.5 years, and 71.4% of patients were women. Hypopituitarism (64.3%), arginine vasopressin deficiency (50.0%), and visual impairment (50.0%) were frequent at presentation. One patient presented with sudden bilateral visual loss and hemodynamic instability, mimicking an apoplexy-like sellar emergency. Breast cancer was the most common primary tumor (28.6%). Serum prolactin levels, available in 11 patients, were uniformly below the range typically expected for macroprolactinoma despite large sellar masses. Histopathologic confirmation was obtained in 35.7% of cases, whereas the remainder fulfilled high-likelihood clinicoradiologic criteria. Median overall survival after pituitary metastasis diagnosis was 6.5 months, and 92.9% of patients died during follow-up.

**Conclusion:**

Pituitary metastasis frequently presents with combined endocrine dysfunction and neuro-ophthalmologic compromise, occasionally as an acute sellar emergency. In oncologic patients with sellar lesions, the combination of AVP-D, visual deterioration, and non-prolactinoma-range hyperprolactinemia should raise suspicion for pituitary metastasis and prompt urgent endocrine and local evaluation.

## Introduction

Pituitary metastases represent a rare but clinically consequential oncologic event, typically arising in the setting of advanced systemic malignancy [1, 2]. Despite their rarity relative to pituitary adenomas, this diagnosis should not be overlooked given the risk of rapid clinical decompensation [3]. Pituitary metastases may present with acute endocrine or neuro-ophthalmologic deterioration, including hypopituitarism, central arginine-vasopressin deficiency (AVP-D), visual symptoms, or cranial nerve palsies, and therefore require timely recognition [4, 5].

However, guidance for day-to-day clinical management remains limited and largely experience-based. population-level datasets provide important epidemiological context, but they seldom capture the bedside phenotype that drives urgent decisions—such as the tempo of visual decline, cranial neuropathies, and the severity of endocrine dysfunction. Conversely, because pituitary metastases are rare, published institutional series are often small and heterogeneous in diagnostic confirmation and treatment approaches, which limits direct comparisons and makes it difficult to develop practical clinical frameworks.

In this study, we aimed to characterize the clinically actionable presentation of pituitary metastasis in a contemporary tertiary-center cohort. We describe presenting manifestations, endocrine dysfunction, radiologic features, primary tumor origins, and outcomes, with particular emphasis on diagnostic clues that may increase bedside suspicion. We further anchor these cohort-level findings in two illustrative clinical vignettes that highlight two relevant clinical scenarios: an acute endocrine–neuro-ophthalmologic emergency that may mimic other sellar crises, and a biologically distinct metastatic presentation in which the significance of sellar involvement is shaped by the underlying primary tumor. Rather than presenting pituitary metastasis solely as a rare oncologic endpoint, we aim to frame it as a recognizable clinical syndrome in which endocrine dysfunction, neuro-ophthalmologic compromise, and cancer context intersect to inform diagnostic suspicion and multidisciplinary evaluation.

## Methods

### Study design and eligibility

We conducted a retrospective, single-center case series of patients with pituitary metastasis.

Patients were eligible if pituitary metastasis was diagnosed between 2010 and 2025 based on histopathology and/or radiologic and clinical criteria in the context of known systemic malignancy.

Exclusion criteria were: (1) sellar/suprasellar lesions not consistent with metastatic disease (e.g., primary pituitary tumors or other non-metastatic sellar pathologies); (2) insufficient clinical, imaging, and/or pathology information to support the diagnosis; and (3) incomplete records precluding ascertainment of key presentation variables and/or survival outcomes.

The diagnosis was confirmed by histopathology (*n* = 5) or considered as highly likely because of underlying advanced malignant disease, rapid progression of the sellar mass, and/ or imaging findings considered atypical for benign sellar lesions (*n* = 9).

### Data collection

We extracted the following variables from medical records: demographics (age, sex), performance status (ECOG - Eastern Cooperative Oncology Group [6]) at pituitary metastasis diagnosis, primary tumor type, interval from primary tumor diagnosis to pituitary metastasis diagnosis, presenting symptoms (visual impairment, headache, cranial nerve deficits), endocrine dysfunction (hypopituitarism, AVP-D), evidence of other intracranial and systemic metastases, treatments (systemic therapy, radiotherapy, surgery/biopsy), survival after pituitary metastasis diagnosis, and outcome at last follow-up.

### Definitions

####  Hypopituitarism evaluation

Hypopituitarism was defined by clinician-documented central hormone deficiencies and/or initiation of pituitary hormone replacement [7, 8]. When laboratory data were available, pituitary hormone deficits were defined using standard endocrine criteria, prioritizing clinically actionable axes: central adrenal insufficiency (low morning serum cortisol in the appropriate clinical context and/or requirement for glucocorticoid replacement), central hypothyroidism (low free T4 with low/normal TSH), and central hypogonadism (low sex steroids with low/normal LH and FSH, interpreted in the clinical context) [7]. Because of the retrospective design and the advanced oncologic setting, endocrine testing, including dynamic stimulation tests, was not uniformly performed; therefore, ascertainment emphasized documented diagnoses, treatment decisions, and available objective laboratory data. AVP-D was defined by clinician-documented AVP-D and/or desmopressin initiation in the setting of polyuria/polydipsia, supported by available biochemical data and/or documented response to desmopressin when available.

#### Visual impairment

Visual impairment was defined as decreased visual acuity and/or a visual field deficit at presentation, as documented by the treating team and corroborated, when performed, by neuro-ophthalmologic assessment. Neuro-ophthalmologic evaluation included best-corrected visual acuity testing and standardized automated perimetry.

####  Radiology features

For patients without histopathologic confirmation (*n* = 9), the diagnosis of pituitary metastasis was considered highly likely when at least two of the following three criteria were simultaneously fulfilled, based on the validated model proposed by Yuzkan et al. [9] : rapid growth on serial MRI, defined as a > 20% increase in maximum tumor diameter within six months; nodular or mass-like expansion of the pituitary stalk; and a known history of malignancy. This combination has been shown to discriminate pituitary metastasis from pituitary tumors with high sensitivity and specificity [9]. In all cases, imaging studies were re-reviewed by an experienced neuroradiologist (MP). Additional radiologic features supporting the diagnosis included cavernous sinus invasion, adjacent bone destruction, optic nerve edema, irregular infiltrative tumor contour, and leptomeningeal enhancement, consistent with locally aggressive sellar disease.

#### Time-to-event definitions

Overall survival (OS) was defined from the date of pituitary metastasis diagnosis to death or last follow-up. OS was estimated using the Kaplan–Meier method. Exploratory subgroup descriptions were performed according to clinically relevant baseline variables, including ECOG performance status and the presence of other intracranial metastases. Given the small sample size and limited statistical power, all survival analyses were considered exploratory and descriptive.

#### Statistical analysis

 We used descriptive statistics. Continuous variables are reported as median (IQR) and categorical variables as n (%). survival was summarized from the pituitary metastasis diagnosis to death or last follow-up.

#### Ethics approval

This study was approved by the Ethics Committee of Hospital das Clinicas da Faculdade de Medicina da Universidade de São Paulo (CAAE: 86575324.3.0000.0068).

## Results

### Cohort characteristics and primary tumors

Fourteen patients with pituitary metastasis were included. Median age at diagnosis was 52.5 years (IQR 40.5–64.3), and 10 patients (71.4%) were women. Breast cancer was the most frequent primary tumor (4/14, 28.6%). In most patients, pituitary metastasis occurred in the setting of advanced systemic malignancy, although the interval between primary tumor diagnosis and pituitary involvement was heterogeneous. t he median interval between primary cancer diagnosis and pituitary metastasis was 6.5 months (range, 0–54 months).

### Clinical and endocrine presentation

Clinical presentation was dominated by combined endocrine and neuro-ophthalmologic dysfunction. Hypopituitarism was present in 9/14 patients (64.3%), arginine vasopressin deficiency (AVP-D) in 7/14 (50%), and visual impairment in 7/14 (50%). Visual impairment was heterogeneous and reflected distinct anatomical patterns of sellar and parasellar involvement. When detailed information was available, presentations included chiasmal visual field defects, decreased visual acuity, bilateral visual loss, ophthalmoplegia, and orbital apex/cavernous sinus syndromes. Case 1 presented with bitemporal hemianopia and progressive worsening of visual acuity during hospitalization; MRI showed involvement of the optic chiasm and optic tracts, and visual improvement was documented after transsphenoidal resection. Case 3 evolved from unilateral ptosis and blurred vision to bilateral ptosis, bilateral amaurosis, and complete bilateral ophthalmoplegia, consistent with extensive skull-base, cavernous sinus, optic canal, and orbital apex involvement; visual loss persisted after radiotherapy. Case 7 developed rapidly progressive bilateral visual loss, initially affecting the left eye and subsequently the right eye, with neuro-ophthalmologic assessment documenting no light perception bilaterally due to optic nerve and chiasmal compression. Case 12 presented with a right orbital apex/cavernous sinus syndrome, including ptosis, mydriasis, third cranial nerve palsy, V1 sensory loss, proptosis, and impaired right visual function; OCT was attempted but could not be obtained in the right eye because of ocular positioning, whereas left-eye OCT showed no abnormalities. Case 14 had decreased visual acuity associated with a suprasellar/hypothalamic lesion compressing and involving the optic chiasm, without documented ocular motor deficit on bedside examination. Headache, cranial nerve dysfunction, and other symptoms of sellar mass effect were variably observed. This pattern supports pituitary metastasis as a clinically actionable syndrome in which endocrine failure and visual compromise frequently coexist at presentation. Serum prolactin levels were available in 11 patients. The median prolactin concentration was 36.3 ng/mL (range, 5.4–132.5 ng/mL). Prolactin levels were above 25 ng/mL in 7 of 11 patients (63.6%), with elevated values ranging from 33.7 to 132.5 ng/mL. Nonetheless, all prolactin values remained below the range typically expected for macroprolactinoma [10, 11]. This profile was more consistent with disconnection hyperprolactinemia than with a lactotroph tumor. Five patients had a 0-month interval between primary tumor diagnosis and pituitary metastasis (Table 1), reflecting synchronous recognition during the same diagnostic episode. In one patient with an unknown primary tumor, visual impairment and AVP-D led to sellar biopsy, which established the diagnosis of metastatic carcinoma and represented the presenting manifestation of an occult malignancy.

**Table 1:** Clinicopathological features, treatment patterns, and outcomes of a 14-patient series of pituitary metastases

Case	Primary tumor	Age	Sex	ECOG	Interval primary→Pit met (mo)	Visual defect	Hypopituitarism	AVP-D	intracranial mets	Other systemic mets	Tx systemic	Tx radiotherapy	Surgery/biopsy	Survival after Pit met dx (mo)	Outcome
1	Skull base squamous cell carcinoma	53	M	3	11	Yes	No	No	Yes	Yes	Yes	Yes	No	3	Deceased
2	Adenoid cystic carcinoma	80	F	1	0	Yes	Yes	No	Yes	Yes	No	Yes	Yes	9	Deceased
3	Parotid gland	44	M	1	1	Yes	Yes	Yes	No	Yes	Yes	Yes	Yes	10	Deceased
4	Small cell lung cancer	66	F	2	0	Yes	Yes	No	No	No	No	No	No	3	Deceased
5	Melanoma	28	F	0	25	No	No	No	Yes	Yes	No	Yes	No	7	Deceased
6	Unknown	60	F	3	0	No	No	No	Yes	Yes	No	No	No	3	Deceased
7	Breast cancer HER2 +	51	F	3	50	No	No	Yes	Yes	Yes	Yes	Yes	No	1	Deceased
8	Breast - HER2 -	34	F	1	42	No	Yes	Yes	Yes	Yes	Yes	No	No	0	Deceased
9	Breast - triple negative	60	F	1	48	No	Yes	No	No	Yes	Yes	Yes	Yes	20	Deceased
10	Breast cancer HER2 +	40	F	2	23	Yes	Yes	Yes	Yes	Yes	No	Yes	Yes	7	Deceased
11	Germ Cell Tumor	27	M	1	0	No	Yes	Yes	No	Yes	Yes	No	No	13	Deceased
12	Unknown	52	F	0	0	Yes	No	Yes	No	No	No	No	Yes	6	Deceased
13	Lung	65	F	2	2	No	Yes	No	No	Yes	Yes	Yes	No	9	Alive
14	Linfoma	82	M	3	54	Yes	Yes	Yes	No	Yes	Yes	No	No	0	Deceased

### Radiologic features and diagnostic confirmation

At diagnosis, the mean maximum diameter of the sellar lesion was 2.6 cm, and the median maximum diameter was 2.2 cm (range, 1.4–4.9 cm). Histopathologic confirmation of the sellar lesion was obtained in 5/14 cases (35.7%). In the remaining 9 patients, the diagnosis was considered highly likely based on clinical context and imaging findings, incorporating clinicoradiologic features proposed by Yuzkan et al. [9], including known malignancy, rapid interval growth when serial imaging was available, and/or nodular or mass-like pituitary stalk involvement. Additional imaging findings supporting metastatic disease included cavernous sinus invasion, bone destruction, optic pathway involvement, irregular infiltrative contours, and leptomeningeal or additional intracranial metastatic involvement when present.

Among the nine patients without histopathologic confirmation of the sellar lesion, three survived longer than 3 months and had follow-up imaging that allowed assessment of sellar lesion response after treatment. In two of these patients, radiologic regression of the pituitary lesion was documented. In the patient with metastatic non-seminomatous germ cell tumor, systemic chemotherapy with etoposide–cisplatin followed by etoposide–ifosfamide–cisplatin was followed by a substantial decline in tumor markers and marked regression of the sellar/suprasellar lesion: the initial 2.0 × 1.3 × 1.3 cm mass was no longer visible as an expansile sellar or suprasellar lesion on follow-up MRI 7 months later. In the patient with metastatic lung adenocarcinoma, after carboplatin–paclitaxel chemotherapy and stereotactic radiotherapy, the sellar lesion decreased from approximately 1.8 × 1.6 × 1.7 cm to 1.7 × 1.0 × 0.5 cm on follow-up MRI. In the third evaluable non-biopsied patient, treated with whole-brain radiotherapy, follow-up imaging showed stability of the sellar lesion. These radiologic responses or stability after oncologic treatment further supported the metastatic nature of the sellar lesions in non-biopsied cases with evaluable follow-up.

**Fig. 1:**
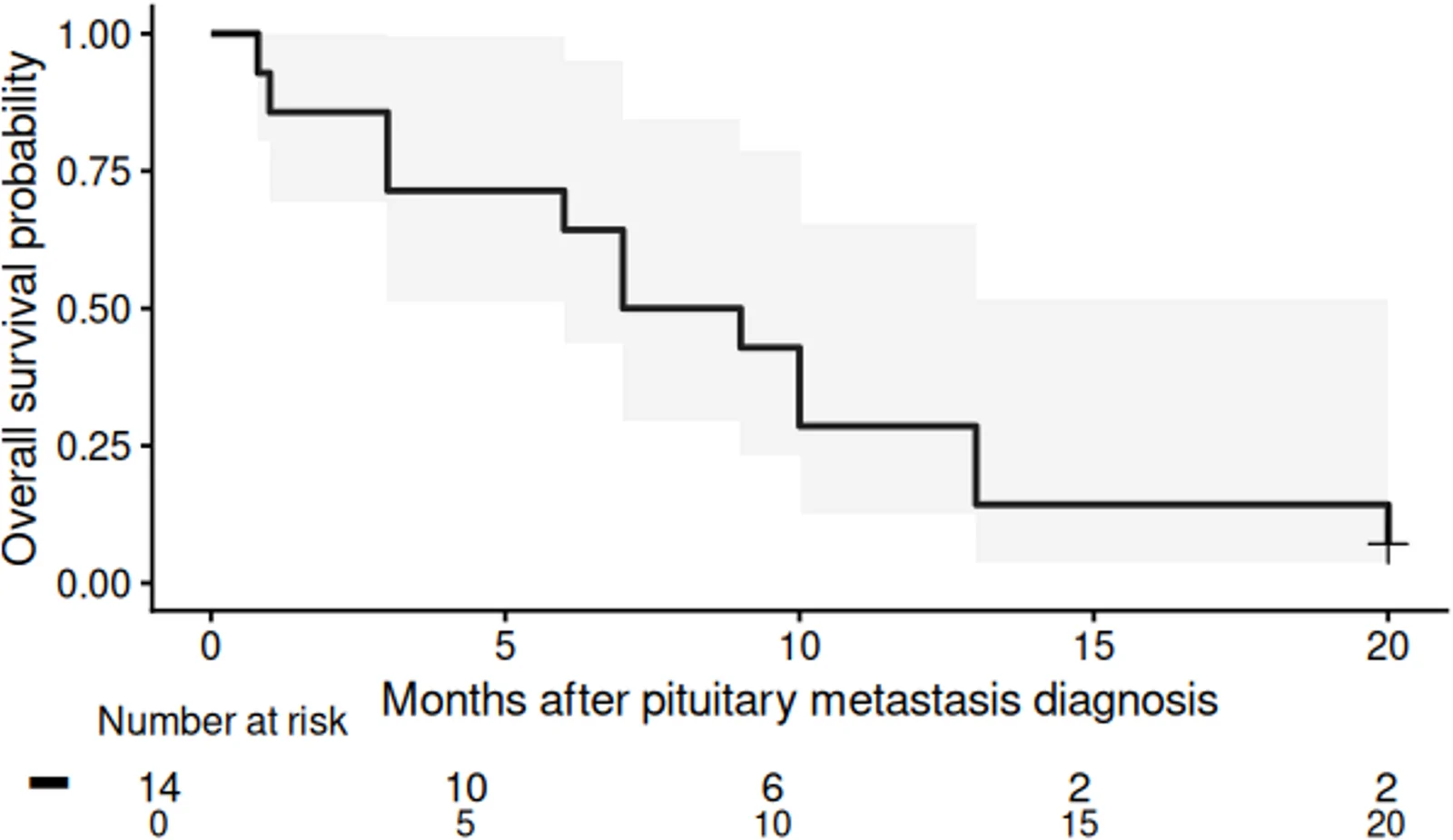
Kaplan–Meier estimate of overall survival after pituitary metastasis diagnosis. Shaded area represents the 95% confidence interval

Among patients with an unknown primary tumor, one underwent biopsy of the sellar lesion, which showed poorly differentiated metastatic carcinoma involving the pituitary gland. Immunohistochemistry showed CK7 positivity, negative pituitary hormones, negative chromogranin and synaptophysin, and a high Ki-67 index of 30%–40%, supporting metastatic carcinoma rather than a pituitary neuroendocrine tumor. In the non-biopsied sellar case, the diagnosis was supported by biopsy-proven hepatic metastatic adenocarcinoma, systemic metastatic disease, additional intracranial lesions, and a large heterogeneously enhancing sellar/suprasellar lesion with mass effect on adjacent neurovascular structures; histological confirmation of the sellar lesion was not available. (Fig. 1)

## Illustrative clinical vignettes

### Clinical vignette 1. Apoplexy-like pituitary metastasis unveiling clinically meaningful receptor switching

**Fig. 2:**
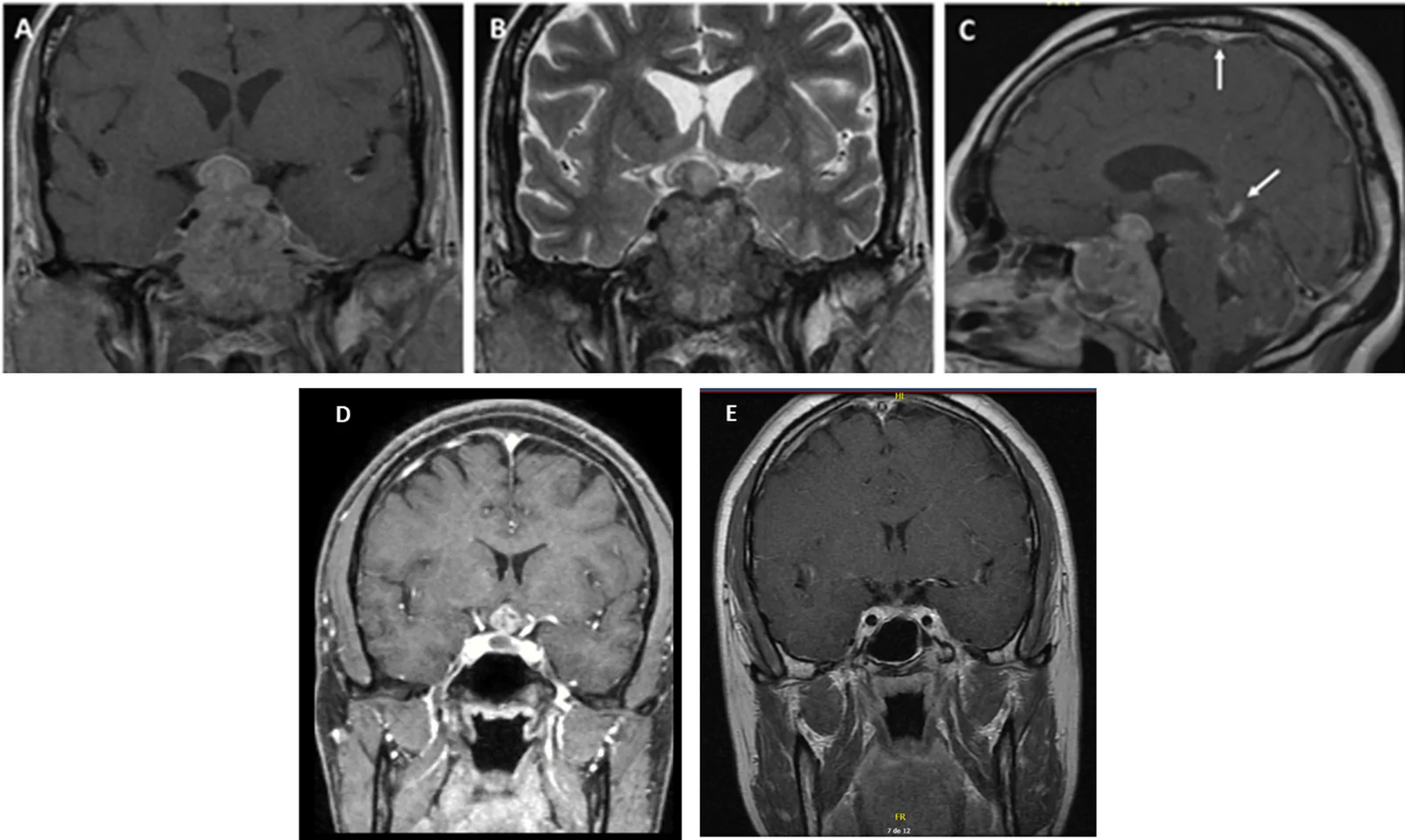
Representative MRI findings from the illustrative clinical vignettes. Case 1 (**A**–**C**). Baseline MRI in a patient with breast cancer presenting with hypopituitarism and AVP-D. (**A**) Coronal contrast-enhanced T1-weighted image demonstrating an expansile sellar lesion with suprasellar extension. (**B**) Coronal T2-weighted image further depicting the lesion. (**C**) Sagittal contrast-enhanced T1-weighted image showing locally aggressive behavior with clival involvement/destruction and suprasellar extension (arrows), supportive of pituitary metastasis. Case 2 (**D**–**E**). Pituitary metastasis from a non-seminomatous testicular germ cell tumor in a patient presenting with hypopituitarism and AVP-D. (**D**) Baseline coronal contrast-enhanced T1-weighted MRI demonstrating a solid-cystic sellar mass with suprasellar extension. (**E**) Follow-up coronal contrast-enhanced T1-weighted MRI after systemic chemotherapy showing marked interval reduction of the sellar lesion, consistent with radiologic response

A 43-year-old woman with a history of invasive micropapillary breast carcinoma previously classified as luminal A developed abrupt bilateral visual loss, severe headache, polyuria, and hemodynamic instability three years after initial treatment, while receiving endocrine therapy with letrozole. MRI revealed an invasive sellar lesion with supra-, para-, and infrasellar extension, optic chiasm compression, and aggressive local features (Fig. 2A–C). Endocrine evaluation showed central adrenal insufficiency, with cortisol 2,5 µg/dL and ACTH < 2.0 pg/mL, and central hypothyroidism, with low free thyroxine and inappropriately low/normal TSH. Gonadotropins were suppressed/inappropriately low. The patient received glucocorticoid replacement and levothyroxine. The pituitary metastasis measured 1.8 × 1.5 × 1.2 cm at diagnosis. Partial transsphenoidal resection confirmed pituitary metastasis from breast carcinoma; importantly, the metastatic lesion had lost ER and PR expression and displayed a triple-negative phenotype, in contrast to the original luminal A primary tumor. The patient subsequently received whole-brain radiotherapy for concomitant meningeal involvement but experienced rapid clinical decline and died three months later. Because of this short clinical course, follow-up sellar MRI to assess post-radiotherapy regrowth was not performed. This vignette illustrates two particularly consequential aspects of pituitary metastasis emphasized in this study: its capacity to present as an acute endocrine–neuro-ophthalmologic emergency closely mimicking other sellar crises, and the potential value of tissue confirmation in revealing biologically meaningful tumor evolution not apparent from the primary tumor alone.

### Clinical vignette 2. Pituitary metastasis from a rare but potentially treatable primary tumor

A 27-year-old man with a non-seminomatous testicular germ cell tumor presented with widely metastatic disease, including pulmonary, nodal, retroperitoneal, and hepatic involvement. Staging brain MRI disclosed a solid-cystic sellar mass with suprasellar extension (Fig. 2D–E). Although he had no visual deficits, endocrine assessment supported hypopituitarism and AVP-D, and hormone replacement was initiated. He then received systemic chemotherapy with etoposide–cisplatin followed by etoposide–ifosfamide–cisplatin, after which tumor markers declined substantially. Follow-up sellar MRI 7 months later demonstrated marked interval regression of the pituitary lesion, from an initial 2.0 × 1.3 × 1.3 cm sellar/suprasellar mass to no visible expansile sellar or suprasellar lesion, consistent with radiologic response. The patient subsequently abandoned systemic therapy and died 13 months after the pituitary metastasis diagnosis. Following treatment discontinuation, no further imaging was performed. The initial responsiveness suggests that in tumors with favorable biology, pituitary metastasis may retain meaningful sensitivity to systemic therapy, and the clinical significance of sellar involvement should be interpreted in light of the underlying primary tumor. (Table 2)

**Table 2:** Demographic characteristics, clinical presentation, and outcomes of patients with pituitary metastasis stratified by the presence of arginine vasopressin deficiency (AVP-D)

	AVP-D	No AVP-D
*n*	7	7
Age, median (IQR)	44.0 (37.0–51.5)	60.0 (56.5–65.5)
Female, n (%)	4 (57.1)	6 (85.7)
Visual defect, n (%)	4 (57.1)	3 (42.9)
Hypopituitarism, n (%)	5 (71.4)	4 (57.1)
Systemic therapy, n (%)	5 (71.4)	3 (42.9)
Radiotherapy, n (%)	3 (42.9)	5 (71.4)
Surgery/biopsy, n (%)	3 (42.9)	2 (28.6)
Primary→Pit met, median (IQR) mo	23.0 (0.5–46.0)	2.0 (0.0–18.0)
OS after Pit met, median (IQR) mo	6.0 (0.5–8.5)	7.0 (3.0–9.0)
Deaths, n (%)	6 (85.7)	6 (85.7)

## Discussion

In this contemporary tertiary-center series, pituitary metastasis emerged not only as a manifestation of advanced systemic malignancy, but also as a clinically recognizable endocrine–neuro-ophthalmologic syndrome. Hypopituitarism, AVP-D, and visual impairment were common at presentation, reinforcing that the bedside relevance of pituitary metastasis lies less in its rarity than in the combination of diagnostic difficulty and potential for rapid clinical deterioration. Within the differential diagnosis of sellar lesions, this pattern matters because it differs from the more indolent and often anterior-pituitary–predominant presentation expected of most non-metastatic pituitary tumors.

The distribution of primary tumors in our cohort was broadly consistent with prior literature, in which breast and lung cancers predominate among the most frequent sources of pituitary metastasis [1, 3, 5, 12, 13]. At the same time, the presence of less common primary tumors, including skull base squamous cell carcinoma, adenoid cystic carcinoma, parotid gland carcinoma, melanoma, germ cell tumor, and lymphoma, as well as two cases with an unknown primary tumor reinforces an important practical point: while knowledge of the most likely oncologic sources is useful for pre-test reasoning, pituitary metastasis should not be excluded solely because the underlying malignancy is unusual [14–17]. In real-world practice, the diagnosis remains anchored not only in tumor origin, but in the convergence of cancer context, clinical tempo, endocrine dysfunction, and imaging pattern.

Among these clues, AVP-D remains particularly informative. Prior series have consistently shown a strong association between pituitary metastasis and posterior pituitary or infundibular involvement, which helps explain why AVP-D is encountered far more often in metastatic sellar disease than in common pituitary adenomas [18, 19]. AVP-D is more frequent in pituitary metastases than in pituitary adenomas, but it is not specific for metastatic disease. Other non-adenomatous sellar or stalk disorders, including hypophysitis, lymphoma, germ cell tumors, Langerhans cell histiocytosis, granulomatous or infectious diseases, and other infiltrative processes, may also present with AVP-D [20]. However, in oncologic patients with sellar lesions, AVP-D should be regarded as a high-yield red flag, especially when accompanied by visual symptoms and a short clinical course.

An additional exploratory observation was that the interval from primary tumor diagnosis to pituitary metastasis appeared longer in patients with AVP-D than in those without it. Although this finding should be interpreted with considerable caution given the very small sample size and heterogeneity of primary tumors, it raises the possibility that clinically overt posterior pituitary involvement may, in some cases, follow a different temporal trajectory from pituitary metastasis without AVP-D. This observation is hypothesis-generating only and requires validation in larger cohorts.

This point becomes even more relevant in the acute setting, where AVP-D may be clinically under-recognized in the presence of concomitant adrenal insufficiency [21]. Glucocorticoid deficiency can mask hypotonic polyuria by impairing renal free-water clearance, such that AVP-D may only become clinically apparent after steroid replacement [22]. For this reason, the absence of overt polyuria at first assessment should not be overinterpreted in unstable patients with suspected sellar metastatic disease. Rather, clinicians should maintain suspicion when the overall endocrine and radiologic pattern suggests posterior pituitary involvement.

The hormonal profile also provides a useful differential clue. In our cohort, prolactin levels were consistently below the range typically expected for macroprolactinoma despite large sellar lesions and, in several cases, substantial suprasellar extension. This pattern strongly favors disconnection hyperprolactinemia rather than autonomous lactotroph secretion. Although mild-to-moderate prolactin elevation may also be seen in non-functioning pituitary tumors with stalk effect [23, 24], the coexistence of such prolactin levels with AVP-D and rapidly evolving neuro-ophthalmologic compromise should heighten suspicion for pituitary metastasis. Stated differently, the combination of AVP-D, visual deterioration, and non-prolactinoma-range hyperprolactinemia appears more informative than any of these features in isolation.

A second clinically important message from this series is that pituitary metastasis may present as a time-sensitive sellar emergency. Visual compromise in metastatic pituitary disease often evolves over days to weeks, but abrupt deterioration can occur, particularly in apoplexy-like presentations or in lesions with hemorrhagic or infarctive change [25]. Our first illustrative vignette was selected because it captures this acute end of the clinical spectrum particularly well: sudden bilateral visual loss, hemodynamic instability, and acute hypopituitarism created a presentation that could easily have been interpreted through a non-metastatic sellar framework. This is precisely why pituitary metastasis must remain in the differential diagnosis of acute visual decline in patients with known malignancy, and also in patients whose clinical or imaging features raise suspicion for an occult cancer. In patients without a known malignancy, the coexistence of AVP-D, rapidly progressive visual or endocrine deterioration, atypical imaging features, and/or additional intracranial or systemic lesions should broaden the differential diagnosis beyond PitNET and raise the possibility of an occult primary malignancy.

The value of tissue confirmation, when feasible, is also illustrated by that same vignette. In that patient, biopsy did more than establish the diagnosis of metastasis: it revealed loss of ER and PR expression and conversion from a luminal phenotype in the primary breast tumor to a triple-negative phenotype at the pituitary site. We believe this observation is important not because it should be generalized from a single case, but because it shows that sellar metastatic tissue may occasionally provide biologically consequential information not evident from the primary tumor alone. This interpretation is consistent with broader evidence from breast cancer brain metastases, in which receptor discordance between primary and metastatic sites is well documented [26]. In our case, pituitary metastasis developed while the patient was receiving letrozole, making endocrine escape biologically plausible. Accordingly, the case supports a measured but clinically relevant point: when tissue is obtained from a pituitary metastasis, biomarker reassessment may add value beyond simple diagnostic confirmation.

The second vignette was chosen for a different reason. Whereas the first underscores acute urgency, the germ cell testicular case highlights that the clinical meaning of pituitary metastasis is not identical across primary tumors. In most patients, pituitary involvement typically occurs in the context of advanced disseminated malignancy and is associated with limited survival, as also reflected in our cohort [12, 27]. However, pituitary metastasis from a non-seminomatous germ cell tumor illustrates that a rare but biologically treatable primary may alter the implications of the sellar lesion. The relevance of this observation is not to suggest that pituitary metastasis is frequently reversible or favorable, but to caution against an overly uniform interpretation of pituitary involvement as a purely terminal event. In selected contexts, tumor biology still matters enough to shape the balance between endocrine support, local symptom control, and systemic treatment intent.

Because histopathologic confirmation is not always feasible in this population, especially in patients with poor performance status or heavy systemic disease burden, the availability of validated clinicoradiologic criteria has practical importance. In our series, most patients were diagnosed without tissue confirmation but fulfilled a high-likelihood clinicoradiologic framework based on malignancy history and characteristic imaging findings. The model proposed by Yuzkan et al. [9] is particularly helpful in this context, as it formalizes what has often been handled informally in practice: rapid sellar growth, nodular or mass-like stalk involvement, and a known history of malignancy substantially increase the probability that a sellar lesion represents metastatic disease rather than a primary pituitary tumor. We view this not as a substitute for pathology when pathology is clinically indicated and safely obtainable, but as a rational tool for diagnostic adjudication in situations where biopsy is not justified or would not change management.

As expected, overall survival after pituitary metastasis diagnosis was short in our cohort, supporting the interpretation of pituitary involvement as a marker of advanced cancer burden in most patients [1, 28, 29]. This finding is consistent with previous reports and should temper overly aggressive causal inferences about the apparent effect of local therapies. Although surgery, biopsy, or radiotherapy may be essential for diagnosis or symptom control in selected patients, survival comparisons in cohorts of this size are highly susceptible to confounding by indication and performance-status bias. For that reason, our data are best interpreted as descriptive rather than as evidence for the superiority of any particular local intervention. The same caution applies to exploratory stratification by clinical variables such as ECOG status or intracranial disease burden: these factors are likely relevant, but our series is too small to support stable prognostic modeling.

This study has several limitations. Its retrospective single-center design imposes the usual risks of incomplete ascertainment and selection bias. The cohort is necessarily small, reflecting the rarity of pituitary metastasis, and primary tumors were heterogeneous. Histopathologic confirmation was not available in all cases, although this limitation partly reflects the real-world clinical context in which tissue sampling is often not feasible or not justified. Detailed neuro-ophthalmologic assessments were not uniformly available because of the retrospective design and advanced oncologic context. OCT imaging of the retina and optic discs was not routinely performed and therefore could not be systematically analyzed. An additional limitation is that, because this series was assembled from a tertiary neuroendocrinology service rather than from a comprehensive institutional registry of all sellar lesions, we were not able to determine the proportion of pituitary metastases among all sellar lesions evaluated at our institution during the study period. This is particularly relevant in our setting, as care pathways for patients with systemic malignancy may also involve a dedicated oncology center within the same institution. These constraints should be acknowledged explicitly, but they do not negate the main contribution of the study, which is clinical rather than interventional.

In summary, our data support pituitary metastasis as a recognizable and clinically actionable syndrome at the intersection of endocrinology, neuro-ophthalmology, and oncology. In oncologic patients with sellar lesions, the combination of AVP-D, rapidly evolving visual compromise, and non-prolactinoma-range hyperprolactinemia should raise suspicion for pituitary metastasis and prompt urgent endocrine and local evaluation. The illustrative vignettes reinforce why this diagnosis matters at the bedside: pituitary metastasis may present as an acute sellar emergency, and in selected cases, tissue confirmation may also uncover biologically meaningful information not predictable from the primary tumor alone.

## Data Availability

No datasets were generated or analysed during the current study.
